# Granulation tissue-type hemangioma in a 6-week-old puppy – a case report

**DOI:** 10.1186/s12917-022-03503-1

**Published:** 2022-12-12

**Authors:** Janina Müller, Manfred Henrich, Johanna Hoogen-Merkel, Svenja Hartung

**Affiliations:** 1 Institute of Veterinary Pathology, Justus-Liebig-University Giessen, Frankfurter Straße 96, 35392 Giessen, Germany; 2 Tierpraxis Schwarzach, Körnersbühnd 4, 77836 Rheinmünster, Germany

**Keywords:** Canine, Granulation tissue-type hemangioma, Vascular malformation

## Abstract

**Background:**

Hemangioma is a well-known neoplasia in veterinary and human medicine. Several subtypes have been described and are distinguished based on their histologic appearance. The classification schemes of hemangiomas in human and veterinary medicine are different, and various purpose-based schemes can be found in the literature.

**Case presentation:**

A six-week-old puppy was presented that suffered from a neoplasia that extended to the musculature of the hind limb. After surgical excision, the mass was submitted for pathohistological examination. The mass was composed of endothelial cells forming vascular slits admixed with a fibrous stroma and spindle cells. Immunohistological examination was positive for factor VIII-related antigen and smooth muscle actin, supporting the diagnosis of hemangioma.

**Conclusion:**

The final diagnosis of granulation tissue-type hemangioma was given due to the histological appearance of the neoplasia. Granulation tissue-type hemangioma is a rare subtype of hemangioma. In this case an uncommonly young dog was affected.

## Background

Vascular tumors are common neoplasms in dogs. Approximately half of all canine vascular tumors occur in the skin, and most of them are benign neoplasms or tumor-like lesions (e.g., vascular malformations) [1]. Hemangiomas are benign tumors of the blood vessels and are a common and well-known lesion in veterinary medicine, especially in dogs, where they account for about 3.8 to 4.5% of all skin tumors [2, 3]. They usually occur in older dogs more than 10 years of age and are uncommon below 3 years of age [4]. In contrast, vascular tumors are more common in horses and calves at a young age [5–7]. In young animals, it is often difficult to distinguish between a benign neoplasm and a vascular malformation (nevus or hamartoma). Apart from UV-induced hemangiomas, the etiology of canine hemangiomas is unknown [3]. There is a suggested breed-specific predisposition in several breeds, such as American Staffordshire Terrier, Beagles and Dalmatians but they are common in light-skinned dogs and are related to UV-induced hemangiomas [3, 4, 8]. Granulation tissue-type hemangiomas (GTH) are rarely described in veterinary medicine. Because the veterinary literature on this subtype is sparse, sex predilection or predilected anatomical sites are unknown.

## Case presentation

**Fig. 1:**
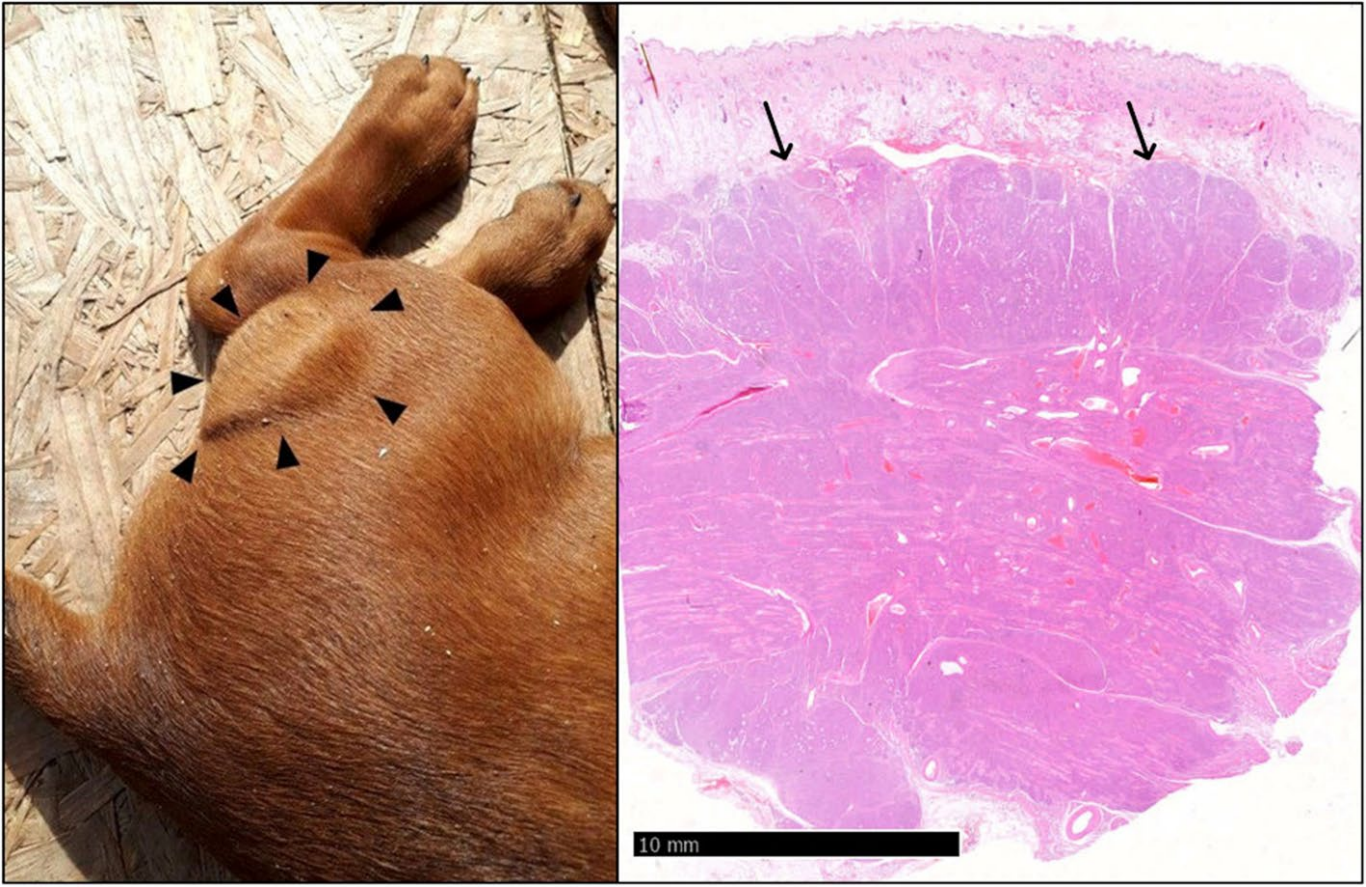
**A** Mass caudolateral on the left hindlimb before excision: 7 × 6 × 1.5 cm subcutaneous proliferation (arrowheads). **B** Hematoxylin and eosin-stain: Note the subcutaneous location and demarcation from surrounding tissue (arrows)

**Fig. 2:**
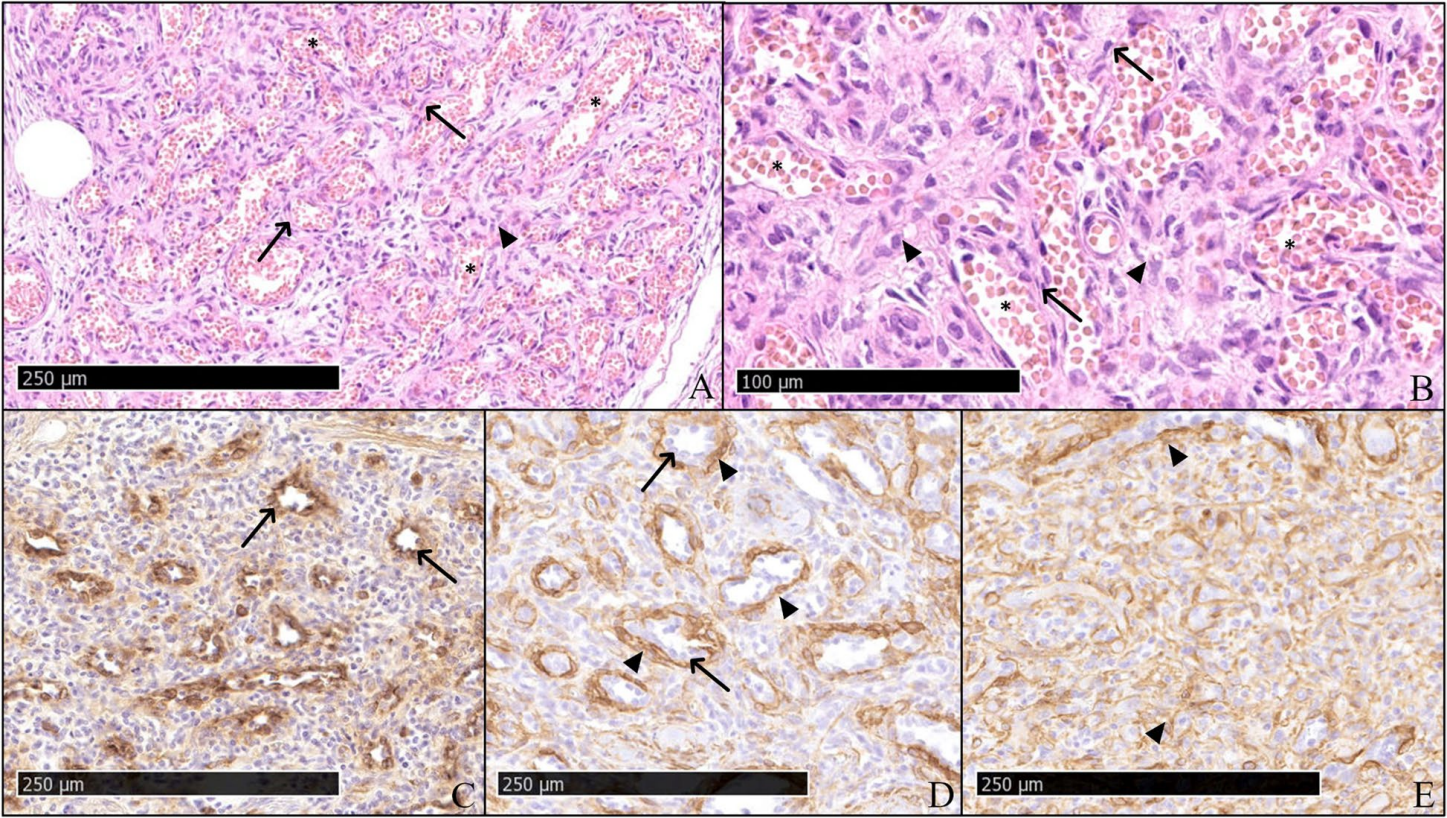
**A**, **B** HE-stain: Split population of plump endothelial cells with a moderate amount of basophilic cytoplasm and a round to oval nucleus with finely stippled chromatin (arrows) build erythrocyte-filled channels (asterisk) as well as spindle cells with a moderate amount of pale basophilic cytoplasm and oval nuclei with mostly one nucleolus and coarsely clumped chromatin with a fibrous stroma surrounding the vascular structures (arrowheads); **C** Factor-VIII-related antigen: Positive signal of endothelial cells (arrows); **D**, **E** SMA: Mainly positive signal of spindle cells (arrowheads) and negative endothelial cells (arrows)

A six-week-old female Hanoverian Scenthound was presented because of a 3 × 4 × 1 cm subcutaneous neoplasm on the left lateral hind limb that was associated with the musculature (Fig. 1A). The regional lymph node (Ln. popliteus) was not affected. At 2 weeks of age, all puppies had been affected by pyoderma. The puppy was initially treated with Traumeel® (Biologische Heilmittel Heel GmbH, Germany). Two days later, the puppy was presented again as no changes occurred. Pulsation was palpable, and sonography showed an inhomogeneous structure rich in blood vessels. Four weeks after the initial presentation, the mass had grown to 7 × 6 × 1.5 cm and had invaded the surrounding tissue. There was no demarcation to the gastrocnemius muscle and flexor digitalis superficialis muscle and a strong blood supply. The mass and regional lymph node were surgically removed at the owner’s decision. Four days after initial treatment, surgical revision and renewal of vessel ligation were performed due to severe postoperative bleeding in the wound area. Subsequently, wound healing proceeded without complications. At the time of writing (5 months postoperatively) the animal displays no evidence of recurrence and the puppy developed physiologically without loss of performance or physical limitations. The mass and lymph node were sent for histopathologic examination. The samples were processed routinely, and sections were stained with hematoxylin and eosin stain. Immunohistochemistry was performed to detect factor VIII-related antigen (rabbit anti-factor VIII PAP polyclonal, Agilent Technologies®, Waldbronn, Germany; formerly DAKO® Hamburg, Germany), alpha-smooth muscle actin (αSMA; mouse anti-alpha-smooth-muscle-actin ABC monoclonal Agilent Technologies®, Waldbronn, Germany), vimentin (mouse anti-vimentin ABC monoclonal, Agilent Technologies®, Waldbronn, Germany), desmin (mouse anti-desmin ABC monoclonal, Agilent Technologies®, Waldbronn, Germany), pan-cytokeratin (mouse anti-cytokeratin pan ABC monoclonal, OriGene Europe®, Herford, Germany) and melan A (mouse anti-melan A monoclonal, Santa Cruz Biotechnology®, Heidelberg, Germany). Biotinylated horse anti-mouse antibody (Vector Laboratories®, Eching, Germany) and biotinylated pig anti-rabbit antibody (Agilent Technologies®, Waldbronn, Germany) were used as secondary antibodies. Histopathology revealed a multinodular, unencapsulated, well-demarcated, partly infiltrative, moderately cellular subcutaneous mass that replaced subcutaneous adipose tissue and skeletal muscle (Fig. 1B). The mass consisted of streams and bundles of two populations of cells. One population of plump endothelial cells with a moderate amount of basophilic cytoplasm and a round to oval nucleus with finely stippled chromatin (Fig. 2A, B) formed slit-shaped vascular channels. The second population consisted of spindle cells with a moderate amount of pale basophilic cytoplasm and oval nuclei with mostly one nucleolus and coarsely clumped chromatin. The spindle cells and a fibrous stroma surrounded the slit vascular channels, occasionally with spindle cells arranged in circular manner around these channels, forming vascular structures (Fig. 2A, B). In some areas, the neoplasm consisted only of spindle cells on a fibrous stroma; in others, the spindle cell population was intermingled with the vascular structures or mostly consisted of vascular structures. There was moderate anisocytosis and anisokaryosis. Mitoses were rare in both populations (< 1 mitosis per high power field (HPF) [0,273 cm^2^]). The tissue was infiltrated with small numbers of lymphocytes, macrophages, and plasma cells. Neutrophilic granulocytes were found in some vascular structures formed by the tumor (not shown). There was mild regeneration of the skeletal musculature. The endothelial cells were positive for factor VIII-related antigen (Fig. 2C), and a subset of the spindle cells, particularly those encircling slit-like vascular channels, were positive for smooth muscle actin (Fig. 2D, E). Both cell populations were positive for vimentin and negative for pan-cytokeratin, desmin and melan A. Follicular hyperplasia, blood resorption and mild sinus histiocytosis were found in the lymph node. The histologic and immunohistological pattern supports the diagnosis of hemangioma. Based on the histological appearance, the neoplasm was classified as a granulation tissue-type hemangioma.

## Discussion and conclusions

In both human and veterinary medicine, several different classifications lead to confusion in the accurate diagnosis of vascular lesions. In particular, the distinction between hemangiomas (benign neoplasms of the vascular system that often occur in elderly patients) and vascular malformations (usually visible in infancy) is fluid, and the subclassification of these tumors is often vague and not clearly defined. The current International Society for the Study of Vascular Anomalies (ISSVA) classification of benign vascular tumors in humans distinguishes infantile hemangioma, congenital hemangioma, tufted angioma, spindle-cell hemangioma, epithelioid hemangioma, and pyogenic granuloma, in addition to some other subtypes that differ in appearance, incidence and prognosis [9]. Infantile hemangiomas in particular have been described to regress spontaneously in 50% of cases by 5 years of age, whereas pyogenic granulomas require treatment up to major, more invasive surgical excision [10, 11]. Spontaneous regression of congenital hemangiomas has also been described in calves [12]. In veterinary medicine, hemangiomas are usually divided primarily into two subtypes: cavernous hemangiomas and capillary hemangiomas [13]. Cavernous hemangioma is the most common type in dogs [1, 14]. Following human medicine, a much more precise classification and evaluation of the tumor is possible: in addition to the above subtypes, a distinction can be made between angiokeratomas, infiltrative growing, arteriovenous, spindle-cell, UV-induced, and granulation tissue-type hemangioma [3]. However, this classification is rarely used in the literature, making a more accurate assessment of prognosis and incidence difficult. According to the above classification, the diagnosis of GTH (also known as lobular capillary granuloma or pyogenic granuloma) can be made based on the histologic and immunohistological picture. GTH is characterized by the formation of capillary-like vessels, sometimes also larger vessels, separated by a fibrous stroma. It is distinguished from granulation tissue by the chaotic arrangement of the vessels and the overall appearance of a neoplasm [3]. The differential diagnoses for this case, based on histopathology and immunohistochemistry, are listed in Table 1. Myofibroblastic tumors are an important and tricky differential diagnosis, especially considering the number of SMA-positive cells between the slit-like vascular channels in the case presented here. The diagnosis is also complicated by the fact that myofibroblasts are an important component of wound healing and may be present in variable numbers in the granulation tissue depending on the maturation rate [15]. The literature on GTH and myofibroblastic tumors contains different definitions and similar features shared by both tumors, making a definitive diagnosis difficult [16–18]. Furthermore, both tumors are described as highly variable in their histologic appearance, and there are only few descriptions in veterinary medicine. Myofibroblastic tumors in animals have mostly been found in the body cavities [18]. Based on gross localization in the skin and the high vascular content confirmed histopathologically, we prefer the diagnosis of GTH. Features of GTH described in the literature are consistent with the histologic picture in this case, and the results of immunohistological examination further support the diagnosis of hemangioma. In human medicine, this subtype is known to be a rare benign vascular tumor mostly located in the skin of the head, the neck or in the oral cavity [19]. In contrast to human medicine, where clustering of hemangiomas is observed in females (with a presumption of a hormonal cause), there is no known sex predilection or predilected locations in dogs [4, 20]. An older study of 208 dogs with vascular tumors found an average of 61% females, suggesting clustering in females and possibly hormonal involvement as in humans [4]. In a study of 126 hemangiomas in dogs, granulation tissue-type hemangioma was found in approximately 10.3%, with no further information on sex or age [21]. Although pyogenic granuloma is classified as a subtype of hemangioma in human medicine, it is often referred to as a vascular malformation rather than a tumor. It frequently occurs in pregnant women, and in addition to a hormonal cause, injury often underlies it [11, 22]. Some human medical studies suggest an association between pyogenic granuloma and Bartonella infection, whereas in veterinary and human medicine cutaneous Bartonellosis is associated with bacillary angiomatosis [23–25]. The presented case is an unusual case of granulation tissue-type hemangioma in a young puppy. In contrast to granulation tissue-type hemangioma in humans this case occurred on the caudal portion of the dog’s body. In dogs, capillary hemangioma also commonly occurs on the dorsal aspect of the limbs but is distinguished from granulation tissue-type hemangioma based on their growth pattern [1]. Most cases of hemangiomas in young children are infantile or congenital hemangiomas. In addition, pyogenic granulomas usually occur in young (pregnant) women, suggesting that hormonal involvement may also be present in dogs. Congenital trauma as a cause cannot be excluded in this case, and a hormonal cause is rather unlikely because of age. In addition, a correlation between the neoplasm and the history of pyoderma in the puppies a few weeks earlier cannot be ruled out. In conclusion, despite the frequency of hemangiomas in dogs, granulation tissue-type hemangioma is a rare subtype that must be kept in mind as a differential diagnosis, especially considering the different classification schemes. Unlike infantile hemangiomas in humans and congenital hemangiomas in humans and cattle, there is no data on possible regression in dogs, so surgical excision should always be favored. A more precise definition of the different subtypes, also in regard to the clinical course, would improve the classification and facilitate the diagnosis. Although all tumors mentioned are considered benign, some subtypes of myofibroblastic tumors with more invasive growth and recurrence have been described in human medicine. To the authors’ knowledge, this is the first published case of granulation tissue-type hemangioma in a young puppy.

**Table 1:** List of possible differential diagnoses with their histologic and immunohistologic features

Entity	Granulation tissue-type hemangioma (GTH) [3, 26, 27]	Granulation tissue [3]	Angiomatosis (secondary to lymphedema)/ Canine Cutaneous Progressive Angiomatosis [3, 28]	Myofibroblast Inflammatory tumor (IMT)/ Inflammatory Pseudotumor (IPT) [16, 17]	Presented case
**Histologic appearance**	- Small branching dilated capillary-type vessels - Larger dilated vessels - Composed of well differentiated endothelial cells - Filled with numerous erythrocytes - Separated by fibrous stroma	- Vessels arranged perpendicular to skin surface	- Increased vessel density between connective tissue and adnexa - Infiltrative growth and not as circumscribed as in GTH - Dilated superficial and hypoplasia of deep lymphatic vessels - All types of vessels may be affected	- Myofibroblast proliferation - Depending on subtypes: - With germinal centre, foamy Macrophages, appearance like fibrous histiocytoma or like desmoid tumor	- Two parts: - Partially composed of small capillary-type vessels and central larger vessels - Partly composed of spindle-cell-population
**Inflamma-tory cells**	- Varying number of inflammatory cells ➔ Degree of inflammatory may depend on stage of development and/or secondary ulceration	- Varying number of inflammatory cells	- Lesser than GTH to no inflammatory cells	- Depending on subtype - Varying number of lymphocytes and plasma cells or histiocytes - 65-90 inflammatory cells/HPF (IMT) - 200-250 inflammatory cells/HPF (IPT)	- Few plasma cells, lymphocytes and macrophages
**Immunohisto-chemistry**	**Positive** - Vimentin, Faktor VIII-related antigen (endothelial cells), SMA (perivascular spindle cells) **Negative** - Desmin, Cytokeratin		- As for vessels, since it resembles “normal” vessel structures	**Positive** - Vimentin, SMA, Desmin - ALK (IMT), IgG4 (IPT) **Negative** - S100, Myoglobin, Cytokeratin	**Positive** - Vimentin, SMA (spindle-cells), Factor VIII (endothelial cells) **Negative** - Desmin, Cytokeratin
**Site of occurrence**	- Skin of head and neck, oral cavity	- Various sites	- Various, mostly skin of the limbs	- Lung, abdominal organs	- Hind limb

## Data Availability

The datasets used and/or analyzed during the current study are available from the corresponding author upon reasonable request.

## Abbreviations

GTH: Granulation tissue-type hemangioma
IMT: Myofibroblast Inflammatory tumor
IPT: Inflammatory Pseudotumor
HE-stain: Hematoxylin and eosin-stain
SMA: alpha-smooth-muscle-actin
